# Advances in Molecular Imaging Strategies for* In Vivo* Tracking of Immune Cells

**DOI:** 10.1155/2016/1946585

**Published:** 2016-09-20

**Authors:** Ho Won Lee, Prakash Gangadaran, Senthilkumar Kalimuthu, Byeong-Cheol Ahn

**Affiliations:** Department of Nuclear Medicine, Kyungpook National University School of Medicine and Hospital, Daegu, Republic of Korea

## Abstract

Tracking of immune cells* in vivo* is a crucial tool for development and optimization of cell-based therapy. Techniques for tracking immune cells have been applied widely for understanding the intrinsic behavior of immune cells and include non-radiation-based techniques such as optical imaging and magnetic resonance imaging (MRI), radiation-based techniques such as computerized tomography (CT), and nuclear imaging including single photon emission computerized tomography (SPECT) and positron emission tomography (PET). Each modality has its own strengths and limitations. To overcome the limitations of each modality, multimodal imaging techniques involving two or more imaging modalities are actively applied. Multimodal techniques allow integration of the strengths of individual modalities. In this review, we discuss the strengths and limitations of currently available preclinical* in vivo* immune cell tracking techniques and summarize the value of immune cell tracking in the development and optimization of immune cell therapy for various diseases.

## 1. Introduction

Immune cells have been studied extensively to elucidate their biological roles under various physiological and pathological conditions. Improved understanding of immune cell functions can help lay the foundation for safe and efficient application of these cells for therapeutic purposes. Moreover, immune cells are being used increasingly as new potential therapeutics to treat conditions such as autoimmune disease and cancer [1]. Noninvasive,* in vivo* cell tracking is an emerging approach for imaging cells in their native environment. Molecular imaging is a rapidly growing field with implications in biology, chemistry, computer science, engineering, and medicine, which allows visualizing cellular and subcellular processes within living subjects at the molecular and the anatomical level [2]. Dynamic noninvasive imaging can direct proper decision-making processes during preclinical and clinical studies, which are aimed at enhancing efficacy and safety of immune cell therapies. Molecular imaging is evolving rapidly and has been facilitated by the development of relevant materials such as imaging agents, reporter constructs, ligands, and probes [3]. Various molecular imaging techniques such as computed tomography (CT), magnetic resonance imaging (MRI), bioluminescent imaging (BLI), fluorescence imaging (FLI), single photon emission computed tomography (SPECT), and positron emission tomography (PET) are actively applied for tracking immune and stem cells [4–9]. Although MRI and CT provide excellent anatomical resolution and are easy to translate into clinical application, these modalities are limited by low sensitivity and high instrumentation cost [10, 11]. CT is one of the radiology technologies applied to track immune cells in the field of biomedical imaging [3, 12, 13]. MRI is now emerging and rapidly expanding wings in the field. It has the advantages of safety, high resolution, and direct applicability to cell tracking in clinical studies [14, 15]. Various types of reporter genes such as those that encode fluorescent and bioluminescent proteins have been used as imaging reporters for visualization and tracking of immune cells* in vivo*. Application of imaging reporters is facilitated by the development of efficient vector delivery systems [3, 9, 16, 17]. BLI can track migration of immune cells to sites of inflammation [18, 19]. FLI has been used in noninvasive* in vivo* tracking of dendritic cell (DC) migration into lymph nodes and primary macrophage migration toward induced inflammatory lesions [4, 20]. PET is a sensitive imaging tool for detecting immune cells in various animal models and provides quantitative and temporal distribution of immune cells by radiolabeling with ^18^F-FDG or ^111^In-oxine [3, 21–25]. The above-mentioned molecular imaging techniques are widely exploited for immune cell monitoring at high resolution in living animals.

Molecular imaging is considered the preferred approach for tracking immune cells in imaging studies* in vivo*. There is therefore a need for researchers to be familiar with proper cell labeling methods and appropriate imaging modalities, specific for the particular labeling method. In this review, we provide a general overview and specific examples of* in vivo* tracking of immune cells, with various imaging modalities for better understanding of the roles played by immune cells under various pathophysiological conditions.

## 2. Advantages and Disadvantages of Each Molecular Imaging Technology

BLI and FLI are relatively low-cost and high-throughput techniques, but they are limited by the lack of fine spatial resolution and difficulty in scaling up for application in larger animals and humans because of inherent depth limitation originating from poor tissue penetration of optical signals [11, 26]. PET and SPECT have the advantages of high sensitivity and unlimited depth penetration, excellent signal-to-background ratios, and a broad range of clinically applicable probes. However, nuclear images have the disadvantages of high background activity and limited anatomical information [27]. Multimodal fusion molecular imaging is now widely applied to overcome the limitations of a single imaging modality. Commercially available systems integrate optical, PET, SPECT, CT, and MRI imaging in various combinations. These multimodal approaches allow different imaging technologies to be combined by simultaneous acquisition and thus together incorporate the best features and utilities of each modality [28].

*In vivo* imaging strategies in preclinical studies have an important advantage: the same animal can be examined repeatedly at different time points, thereby decreasing the variability in study population and reducing the sample size [29, 30]. To monitor adoptively transferred immune cells, an effective labeling methodology needs to be selected. Cell labeling can be classified as either direct or indirect [31]. Direct labeling of the imaging moiety of therapeutic cells is the most commonly used strategy for monitoring cells in living subjects [32]. In direct labeling, the cells can be harvested and labeled with radioisotopes, MRI-based contrast agents, or fluorophores, thereby allowing cells to be visualized by PET/SPECT, MRI, or FLI, respectively. This strategy has the advantages of simple labeling protocols and high sensitivity. However, it has major drawbacks [1]. First, the extent of labeling depends on the ability of the signal element in the cells to retain the label. Second, it does not allow long-term monitoring of cell viability. Proliferation of labeled cells in the living subject results in diluted signal, and persistent signals are emitted from the labeled cells even after cell death. In contrast, indirect labeling with reporter genes such as luciferase (Luc), green fluorescent protein (GFP), and sodium iodide symporter (NIS) does not have such limitations, and this approach is therefore preferred for long-term* in vivo* cell monitoring [33]. In indirect labeling, the cells are transfected with a vector containing the imaging reporter genes. The reporter genes are integrated into the cell genome and transcribed to mRNAs, which are translated to reporter proteins. In stably transfected cells, the reporter gene is inherited by both daughter cells upon cell division. This strategy is essential for long-term* in vivo* tracking of cells and for evaluation of division of labeled cells. Despite the advantages of the indirect cell labeling strategy, it has its own limitations. It is difficult to generate stably transfected cells because of the low efficiency of transfection in immune and primary cells. Safety concerns arising from genetic modification of cells by indirect labeling are an issue that substantially limits clinical application.

## 3. Relevance of Immune Cell Tracking

Tracking of immune cells such as T cells, natural killer cells, DCs, and macrophages is used to develop cell-based immunotherapy approaches against various diseases, primarily malignant diseases [34, 35]. Most of the information about immune cell tracking was previously obtained using flow cytometry and confocal microscopy [36]. Flow cytometry is a good experimental approach for counting transferred immune cells in an organism. However, this is only applicable in the case of* ex vivo* samples and does not provide information about the precise location of the analyzed immune cells. Confocal microscopy can provide information about the spatial distribution of cells by using immunostained tissue sections and real-time* in vivo* distribution of cells in a superficial organ that can be accessed by a light signal. However, confocal microscopy is unsuitable for real-time* in vivo* monitoring of the cells in deep organs.

Recent advances in imaging technology* in vivo* have revealed the potential of various imaging techniques for monitoring immune cells. The functional changes associated with the death, survival, proliferation, and migration of cells can be accurately assessed [37]. Successful application of such* in vivo* immune cell tracking tools can potentially optimize image-guided therapeutic options and eventually may improve therapeutic options or therapeutic outcome. In particular, the best route of administration of therapeutic cells and the optimal dose for cell therapy can be easily determined by imaging.

### 3.1. Immune Cells

#### 3.1.1. Dendritic Cells

Dendritic cells (DCs) occupy a central position in the immune system. DCs are professional antigen-presenting cells (APCs) that play a critical role in the regulation of adaptive immune response [21, 38]. They arise from bone marrow precursors and are present in immature forms in the peripheral tissues. DCs capture and process antigens and then undergo maturation [39]. Mature DCs can stimulate helper and killer T cells* in vivo* by expressing at high levels MHC class I/II molecules, costimulatory molecules (B7), and adhesion molecules (ICAM-1, ICAM-3, and LFA-3) [40, 41]. When used to vaccinate cancer patients, DCs loaded with tumor-associated antigens are a potentially powerful tool for inducing antitumor immunity [42]. Because of these important DC characteristics, many recent studies have tracked DC migration with various imaging modalities. de Vries et al. monitored the migration of antigen-pulsed DCs to the lymph nodes in melanoma patients with gamma camera imaging. They isolated DCs from peripheral blood mononuclear cells (PBMCs) and labeled them with ^111^In-oxine [43]. Olasz et al. tracked the migration of DCs into the lymph nodes with PET imaging modality in the case of bone marrow-derived DCs (BMDCs) labeled with ^18^F-succinimidylfluorobenzoate (SFB) [44]. Noh et al. studied BMDC migration into the lymph nodes by labeling BMDCs with near-infrared- (NIR-) emitting quantum dots (QD) and tracking the labeled cells up to 3 days after injection by using FLI [4]. Kim et al. established DCs expressing ferritin heavy chain (FTH) as an MR reporter gene and monitored DC migration by MRI [45]. Xu et al. successfully labeled mature BMDC with SPIO nanoparticles and monitored BMDC migration* in vivo* toward popliteal lymph nodes by clinical 3T MR scanner [46]. Lee et al. also demonstrated DC migration into lymph nodes with BLI and ^124^I PET/CT imaging modalities using DCs expressing firefly luciferase (Fluc) and sodium iodine symporter (NIS) reporter genes [47] (Figure 2). For clinical application, another study performed evaluation of* in vivo* labeled DC migration in patients with melanoma or renal carcinoma. They generated DCs from PBMC and then labeled immature (i) and mature (m) DCs with radioisotopes ^99m^Tc-HMPAO and ^111^In-oxine, respectively. The results showed that mDCs give approximately 6–8-fold higher uptake in lymph node than immature DCs, and better migration activity was obtained with intradermal administration than with a subcutaneous route [48]. Thus, these studies using various molecular imaging techniques will help evaluate DC-based immunotherapy aimed at increasing the efficacy of DC migration and improving the design of clinical trials (Table 1).

#### 3.1.2. Macrophages

**Figure 1:**
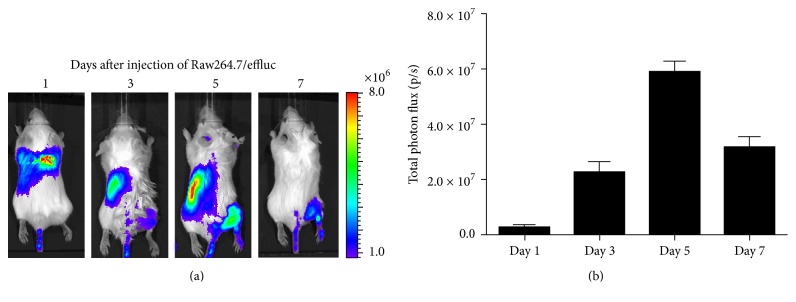
*In vivo* monitoring of macrophage migration toward inflammatory lesions by using optical imaging modality. (a) The right hind limb of Balb/c mice was intramuscularly injected with turpentine oil to induce inflammation. Seven days later, Raw264.7 cells expressing the enhanced firefly luciferase (effluc) gene were intravenously administered to these mice. Bioluminescence imaging was undertaken at days 1, 3, 5, and 7 after injection of Raw264.7/effluc cells. (b) The bioluminescence signals from Raw264.7 cells were used to quantify the migration of cells toward the inflammatory lesion. Data are expressed as the mean ± SD.

**Figure 2:**
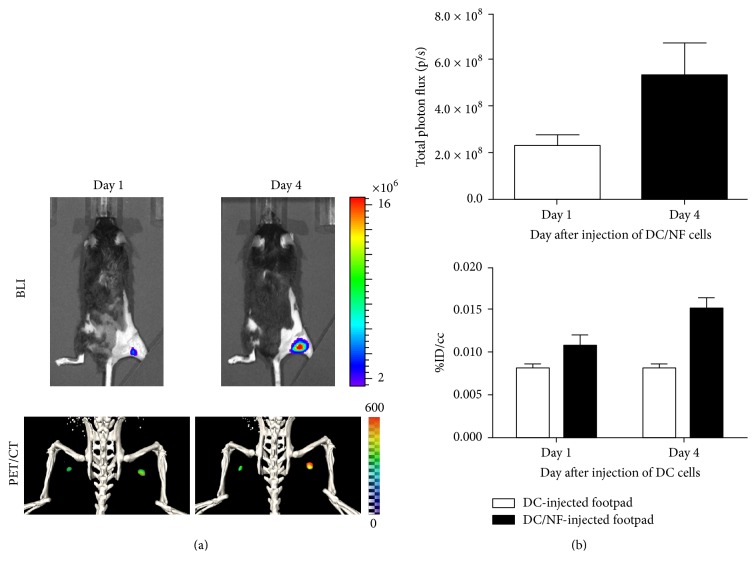
Visualization of DC migration into the lymph node* in vivo* using multimodal imaging. DC2.4 or DC2.4 cells expressing NIS and effluc genes (DC/NF) were injected in the left or right mouse footpad, respectively. (a) Signals were observed in the lymph node by both BLI and ^124^I PET/CT imaging. (b) Quantification of BLI signals and radioiodine uptake in the lymph node. Data are expressed as the mean ± SD.

**Figure 3:**
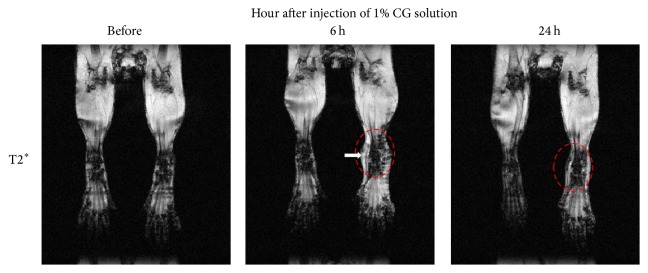
*In vivo* tracking of peritoneal macrophage migration toward CG-induced inflammatory lesion by MRI. Peritoneal macrophages were isolated from C57BL/6 mice at day 4 after injection with 3% thioglycollate medium and then labeled with magnetic nanoparticles. MRI was obtained before or after injection of 1% carrageenan by 4.7T MRI. Arrow indicates hypointense signal of migrated peritoneal macrophages.

Macrophages play crucial and distinct roles in host defense. They are strategically located throughout the body tissues, where they ingest and process foreign materials, dead cells, and debris and recruit additional macrophages in response to inflammatory signals [65–67]. There are two major macrophage subsets: classically activated macrophages (M1) and alternatively activated macrophages (M2 or tumor-associated macrophages, TAMs). The M1 macrophages secrete proinflammatory cytokines such as IL-1*β*, TNF-*α*, IL-6, and IL-12, as well as nitric oxide (NO). They have various functions, including boosting inflammation, debris removal, sterilization, and apoptotic cell removal. The alternatively activated M2 macrophages can be classified into subtypes M2a, M2b, M2c, and M2d which are involved in tissue repair/wound healing and immunoregulatory and immunosuppressive activities [68, 69]. Monitoring of macrophages is necessary to understand inflammatory diseases and tumor microenvironments; therefore, macrophages have been widely investigated using various molecular imaging techniques. Several studies reported successful* in vivo* monitoring of macrophages transfected with reporter genes such as Fluc or NIS in animal models with inflammatory lesions or tumors (Figure 1) [51–53]. Lee et al. investigated the recruitment of iron oxide-labeled primary macrophages to the inflammatory lesion in a mouse model using MRI (Figure 3) [70]. Kang et al. tracked migration of primary macrophages toward carrageenan-induced inflammatory lesions by both FLI and MR with NIR fluorescent magnetic nanoparticles [20]. Gramoun et al. demonstrated tracking of SPION-labeled macrophages using MR to assess treatment effects in a mouse model of rheumatoid arthritis by using MR [50]. TAMs have been successfully monitored with various imaging modalities. Choi et al. reported evaluation of TAM migration into tumor lesions and the modulation of tumor progression using multimodal optical reporter gene imaging [52]. Blykers et al. tracked TAMs using PET/CT with ^18^F-labeled camelid single-domain antibody fragments to target mannose receptor-expressing macrophages using PET/CT [54]. Daldrup-Link et al. showed that SPIO with 2T MRI could be applied to track TAMs in a mouse model of mammary carcinogenesis [49]. Improved understanding of the roles of macrophage migration in inflammation and tumor formation can offer useful clues to modulate macrophage activity by developing and evaluating anti-inflammatory or antitumor compounds.

#### 3.1.3. T Cells

T cells are lymphocytes that play crucial roles in cell-mediated immunity. They have unique surface proteins known as T-cell receptors (TCRs), a complex of integral membrane proteins that recognize antigens when the antigen is presented on the surface of antigen-presenting cells including macrophages, B cells, and DCs [71–73]. Activation of T cells is induced by the interaction between TCR and antigen peptide. There are two main classes of T cells: helper T cells (Ths or CD4^+^ T cells) and cytotoxic T cells (CTLs or CD8^+^ T cells). The Ths recognize the peptides bound to MHC class II molecules. They not only help to stimulate B cells to release antibodies and macrophages to destroy ingested microbes but also help to activate CTLs [74, 75]. On the other hand, CTLs are able to recognize peptides presented by MHC class I molecules and then release cytolytic mediators such as perforin and granzyme, which subsequently induce apoptosis in tumor cells and virus-infected cells [76, 77]. Although T cells possess remarkable potential as a component of immune cell therapy, the fate of the infused T cells and the intermediate steps between cell migration and therapy outcome are not well understood. Many researchers have attempted* in vivo* tracking of the infused T cells with various imaging modalities to determine their biodistribution, viability, and functionality. Chewning et al. generated transgenic mice (T-Lux) in which the luciferase gene is expressed by T cells; T cells were isolated from splenocytes of the T-Lux model mice. Using the BLI imaging system, they visualized T-cell migration to secondary lymphoid tissues within 24 h of adoptive transfer of T-Lux T cells [56]. Kim et al. investigated the targeted movement of CTLs into B-cell lymphomas using BLI [57]. T cells labeled with nanosized MRI contrast agent were observed by MRI to be involved in the rejection of allograft-transplanted hearts and lungs [78]. T-cell migration into melanomas with or without antigen-pulsed DCs was successfully imaged using reporter gene technology combined with PET/CT acquisition, showing increased uptake by the spleen and lymph node with combined immunotherapy, compared to the control [58]. Srinivas et al. visualized T-cell homing behavior in an adoptive transfer model of an autoimmune disease. They labeled T cells isolated from splenocytes of TCR transgenic mice with perfluoropolyether (PFPE) nanoparticle tracer agent and were able to demonstrate* in vivo* T-cell homing to pancreas in a murine diabetes model by ^19^F MRI [55]. Overall, tracking of T cells* in vivo* is useful to understand T-cell biology in various pathophysiological conditions such as autoimmune disorders, cancer, allergy, and transplantation. T-cell tracking will help optimize adoptively transferred T-cell therapy for various disorders.

#### 3.1.4. B Cells

B cells play a vital role in the adaptive immune response to infectious diseases by producing specific antibodies to the antigens expressed by invading pathogens [79, 80]. Antigen-specific interactions require antigens, either free-floating or presented by APCs, to first be internalized by the B-cell receptors (BCR), followed by triggering of signaling cascades that initiate the activation of B cells into antibody-secreting effector cells [81–83]. There are five isotypes of antibodies (IgA, IgD, IgE, IgG, and IgM) based on the C-terminal regions of heavy chains. Antibodies can neutralize infectious pathogens and activate macrophages and other immune cells [84, 85]. Beneficial functions apart, B cells also play a pathological role in allergic and autoimmune diseases, including asthma, rheumatoid arthritis, systemic lupus erythematosus, and vasculitis [86–88]. The use of imaging modality-based tracking of B cells is still in its infancy when compared to tracking of other immune cells. Only a few studies using radioisotopes and magnetic nanoparticles have been reported to date. Walther et al. monitored B cells in the spleen, lymph nodes, testes, and joints by using PET/CT after injecting ^89^Zr-labeled anti-B-cell antibody [60]. Thorek et al. tracked the migration of primary murine B cells toward the spleen by using FLI and MRI with fluorescent and magnetic nanoparticles, respectively [59]. These results can lead to improved understanding of B-cell-related diseases and effective treatment regimens for such diseases.

#### 3.1.5. Natural Killer Cells

Natural killer (NK) cells are lymphocytes of the innate immune system that control several types of tumors and microbial infections [89, 90]. NK cells are regulated by inhibitory/activating receptors, which decide the fate of NK cell [91, 92]. NK cells are activated by interferons (INF-*α*, -*β*, and -*γ*) or macrophage-derived cytokines (IL-12 and IL-18), which results in secretion of cytotoxic granule proteins (perforin/granzyme) that induce apoptosis in target cells [93–95]. Unlike T cells, the non-MHC-restricted cytotoxicity of NK cells renders them appealing for investigation as potential effectors of immunotherapy. Although many studies have investigated the therapeutic effects of NK cell-based immunotherapy for various cancers, alteration of NK cell functions and cytokine imbalance reduce the therapeutic potency of cell therapy [63]. Using NK cells to target malignant cells is another key factor for successful therapy. The tracking of NK cells with various imaging modality techniques can provide information about the presence, quantity, and distribution of administered NK cells in living subjects. For tracking NK cells with nuclear imaging modalities, NK cells were labeled with ^18^F or ^11^C for PET imaging and with ^111^In for SPECT, and the signals emitted from labeled NK cells were observed in lung, spleen, liver, and tumor lesions [62, 64, 96–98]. NK cell tracking with optical imaging modalities was also successfully performed by labeling the NK cells with fluorescent dyes or transfecting with GFP or luciferase reporter genes [61, 99]. Daldrup-Link et al. observed increased fluorescent signal in tumors 24 h after injecting NK cells labeled with DiD (1,1′-dioctadecyl-3,3,3′,3′-tetramethylindodicarbocyanine) fluorescent dye [61]. To track NK cells using MRI, Daldrup-Link et al. transduced NK cells with scFV (FRP5)-zeta and then labeled them with ferucarbotran. The genetically engineered NK cells were injected into NIH 3T3 HER2/neu receptor positive tumor bearing mice, and the group demonstrated increased tumor targeting of the genetically engineered NK cells by 1.5T MR scanner [100]. NK cell tracking might be invaluable for improving the efficacy of NK cell-based immunotherapy by modulating the therapeutic protocols used in translational and clinical approaches.

## 4. Conclusion

*In vivo* tracking of immune cells (DCs, macrophages, T cells, B cells, and NK cells) using various imaging techniques continues to contribute to improved understanding of the role of each immune cell type as well as aiding the development of therapy using or targeting immune cells. Cell labeling, a prerequisite for cell tracking, can be achieved directly or indirectly. Direct labeling strategies using clinically approved materials and methods hold great potential for clinical application. Meanwhile, indirect labeling strategies with reporter genes can assist long-term study of cell survival, proliferation, and activation of immune cells. However, none of the available cell labeling strategies meets all requirements; therefore, an appropriate specific labeling strategy should be selected for each experimental setting.

## Figures and Tables

**Table 1:** Immune cell tracking imaging strategies.

Types of cells	Labeling strategy	Imaging modality	Labeling method	Subject	Duration of tracking	Purpose	Clinical translation	Reference
DC	Direct	FLI	NIR-QD	Mouse	3 days	Tracking study	Limited	[4]
		PET	^18^F-SFB	Mouse	4 h	Tracking study	Yes	[44]
		SPECT	^111^Indium	Human	24–48 h	Tracking study	Yes	[43]
		SPECT	^111^Indium/^99m^Tc-HMPAO	Human	48–72 h	Tracking study	Yes	[48]

DC	Indirect	BLI	Fluc	Mouse	4 days	Tracking study	Limited	[47]
		PET	NIS/^124^I	Mouse	4 days	Tracking study	Yes	[47]
		MRI	FTH	Mouse	48 h	Tracking study	Yes	[45]

Macrophage	Direct	FLI	NIR nanoparticle	Mouse	3–24 h	Tracking to inflammation	Limited	[20]
		MRI	SPIO	Mouse	24 h	Tracking to inflammation	Yes	[49]
		MRI	Magnetic nanoparticle	Mouse	3–24 h	Tracking to inflammation	Yes	[20]
		MRI	SPIO	Mouse	6–13 days	Tracking to rheumatoid arthritis	Yes	[50]

Macrophage	Indirect	BLI	Fluc	Mouse	0–21 days	Tracking to inflammation	Limited	[51]
		BLI	Fluc	Mouse	1–4 days	Colon tumor targeting	Limited	[52]
		PET	NIS/^124^I	Mouse	7 days	Tracking to inflammation	Yes	[53]
		PET	NIS/^124^I	Mouse	8–21 days	Tracking to inflammation	Yes	[51]
		PET/CT	^18^F-FB	Mouse	3 h	Tracking to lung carcinoma	Yes	[54]

T cells	Direct	MRI	IOPC-NH^2^	Rat	24–48 h	Tracking study	Yes	[49]
		MRI	PFPE/^19^F	Mouse	48 h	Tracking study	Yes	[55]

T cells	Indirect	BLI	Fluc	Mouse	24 h	Tracking study	Limited	[56]
		BLI	Fluc	Mouse	10 days	Tracking to lung carcinoma	Limited	[57]
		PET/CT	sr39tk/^18^F-FHBG	Mouse	1–21 days	Melanoma tumor targeting	Yes	[58]

B cells	Direct	FLI	NIR nanoparticle	Mouse	1–15 days	Tracking study	Limited	[59]
		PET/CT	^89^Zr-anti-B220	Mouse	15–72 h	Biodistribution study	Yes	[60]

B cells	Indirect	MRI	SPIO	Mouse	1–15 days	Tracking study	Yes	[60]

NK	Direct	FLI	NIR dye	Rat	24 h	Tracking study	Limited	[61]
		PET	^11^C	Mouse	0.5–1 h	Tracking study	Yes	[62]
		SPECT	^111^In	Human	0.5–144 h	Tracking and therapy study	Yes	[63]
		SPECT	^111^In	Human	6 days	Biodistribution study	Yes	[64]
		SPECT	^111^In	Human	6–96 h	Tracking study	Yes	[63]

DC: dendritic cell, NK: natural killer cell, FLI: fluorescence imaging, PET: positron emission tomography, SPECT: single photon emission computerized tomography, BLI: bioluminescence imaging, MRI: magnetic resonance imaging, CT: computed tomography, NIR: near infrared, QD: quantum dot, SFB: fluorobenzoate, NIS: sodium iodide symporter, HMPAO: hexamethylpropyleneamineoxime, SPIO: superparamagnetic iron oxide, Fluc: firefly luciferase, FB: fluorobenzene, IOPC: iron oxide nanoparticles coated, PFPE: perfluoropolyethers, sr39tk: mutant type of HSV-thymidine kinase, and FHBG: fluorohydroxymethyl butyl guanine.
